# Simple Genome Editing of Rodent Intact Embryos by Electroporation

**DOI:** 10.1371/journal.pone.0142755

**Published:** 2015-11-10

**Authors:** Takehito Kaneko, Tomoji Mashimo

**Affiliations:** 1 Institute of Laboratory Animals, Graduate School of Medicine, Kyoto University, Kyoto, 606–8501, Japan; 2 Institute of Experimental Animal Sciences, Faculty of Medicine, Osaka University, Osaka, 565–0871, Japan; Ohio State University Comprehensive Cancer Center, UNITED STATES

## Abstract

The clustered regularly interspaced short palindromic repeat (CRISPR)/CRISPR-associated (Cas) system is a powerful tool for genome editing in animals. Recently, new technology has been developed to genetically modify animals without using highly skilled techniques, such as pronuclear microinjection of endonucleases. Technique for animal knockout system by electroporation (TAKE) method is a simple and effective technology that produces knockout rats by introducing endonuclease mRNAs into intact embryos using electroporation. Using TAKE method and CRISPR/Cas system, the present study successfully produced knockout and knock-in mice and rats. The mice and rats derived from embryos electroporated with Cas9 mRNA, gRNA and single-stranded oligodeoxynucleotide (ssODN) comprised the edited targeted gene as a knockout (67% of mice and 88% of rats) or knock-in (both 33%). The TAKE method could be widely used as a powerful tool to produce genetically modified animals by genome editing.

## Introduction

Many types of genetically modified (GM) animals have been produced to study human diseases [1–3]. Mice and rats have been used widely as important human diseases model animals [4–6]. Engineered endonucleases, including zinc-finger nucleases (ZFN), transcription activator-like effector nucleases (TALEN) and the clustered regularly interspaced short palindromic repeat (CRISPR)/CRISPR-associated (Cas) system, are recently developed high-impact technologies for the production of GM animals [7–12]. Engineered endonucleases have made the rapid production of GM animals possible without using embryonic stem (ES) cells and induced pluripotent stem cells. However, microinjection of endonucleases into pronuclear-stage embryos is still used routinely, which requires a high skill level to reduce cell damage, and injecting endonucleases into embryos one by one using a micromanipulator is time consuming. Thus, this process has prevented the rapid production of GM animals. Recently, we developed a new technology to produce GM animals without using microinjection of endonucleases into embryos. Technique for animal knockout system by electroporation (TAKE) method [13] is a simple and effective technology that can produce knockout rats by the introduction of endonuclease mRNAs into intact embryos using a three-step electrical pulse electroporation. Electroporation of ZFNs resulted in an embryonic survival rate (91%) and a genome-editing rate (73%) that was more than 2-fold higher than the corresponding rates from conventional microinjection [13]. Previous study reported that the knockout rats were also obtained by electroporation of the CRISPR/Cas system. However, suitable conditions must be demonstrated for the production of knockout and knock-in in rats and other species. This study determined the conditions suitable for the efficient production of knockout and knock-in mice and rats using the TAKE method and the CRISPR/Cas system.

## Materials and Methods

### Animals

C57BL/6J Jcl mice and Jcl:Wistar rats were purchased from CLEA Japan Inc. (Tokyo, Japan). Male mice aged older than 11 weeks and female mice aged from 8 to 16 weeks were used as sperm and oocyte donors, respectively. Female Jcl ICR mice and Jcl:Wistar rats from 8 to 16 weeks old (CLEA Japan Inc.) were used as recipients for embryo transfer. All animals were maintained in an air-conditioned (temperature 24 ± 2°C, humidity 50 ± 10%) and light-controlled room (illuminated from 07:00 to 19:00). All the animal care and procedures performed in this study conformed to the Guidelines for Animal Experiments of Kyoto University, and were approved by the Animal Research Committee of Kyoto University.

### Preparation of Cas9 mRNA, gRNA and ssODN

The plasmid expressing hCas9 (ID#41815) was obtained from the Addgene repository (www.addgene.org/CRISPR), and was modified by addition of the T7 promoter and DNA encoding an SV40 nuclear localisation signals at the N-terminal of hCas9, using an In-Fusion HD cloning kit (Takara Bio Inc., Shiga, Japan), according to the manufacturer’s protocol. Cas9 mRNA was transcribed *in vitro* using an mMESSAGE mMACHINE T7 Ultra Kit (Thermo Fisher Scientific Inc., Waltham, MA, USA) and was polyadenylated using an A-Plus^™^ Poly(A) polymerase tailing kit (Epicentre, Madison, WI, USA). RNA was then purified using a MEGAClear^™^ kit (Thermo Fisher Scientific Inc.).

gRNA was designed to target exon 2 of the mouse and rat interleukin 2-receptor gamma (*Il2rg*) gene (S1 Fig) [14,15]. gRNAs expression plasmids were transcribed *in vitro* using a MEGAshortscript T7 Transcription Kit (Thermo Fisher Scientific Inc.). RNA was then purified using a MEGAClear^™^ kit (Thermo Fisher Scientific Inc.). The sequences of the single-stranded oligodeoxynucleotide (ssODN), which were obtained from IDT (Integrated DNA Technologies Inc., Coralville, IA, USA), are shown in S1 Table.

Cas9 mRNA/gRNA/ssODN for electroporation were resuspended in phosphate buffered saline (PBS with calcium and magnesium free) at 400/600/300, 200/200/200 and 100/100/100 μg/mL, respectively.

### Collection of embryos

Mouse and rat pronuclear-stage embryos were collected using the method described previously by Kaneko [16]. Mouse pronuclear-stage embryos were obtained using an *in vitro* fertilization technique. Cauda epididymis sperm were collected from mature males that euthanized by CO_2_ and cervical dislocation. Sperm were then pre-incubated for 1 h at 37°C under 5% CO 2 and 95% air in 200 μl drops of fertilization medium covered with sterile mineral oil to induce capacitation. Superovulation was induced in female mice by an intraperitoneal injection of 7.5IU pregnant mare’s serum gonadotropin (PMSG) (ASKA Pharmaceutical Co. Ltd., Tokyo, Japan), followed by an intraperitoneal injection of 7.5 IU human chorionic gonadotropin (hCG) (ASKA Pharmaceutical Co. Ltd.) 48 h later. Cumulus-oocytes complexes were collected from oviducts of females that euthanized by CO_2_ and cervical dislocation at 13–15 h after hCG injection. These were then transferred in another 200 μl drops of fertilization medium covered with sterile mineral oil. Capacitated sperm that pre-incubated for 1 h were added into HTF medium drops with oocytes. The oocytes were then co-cultured with sperm at 37°C under 5% CO 2 and 95% air. Pronuclear-stage embryos were collected at 6 h after insemination.

Rat pronuclear-stage embryos were produced by natural mating. Female rats were induced to superovulate by an intraperitoneal injection of 300 IU/Kg PMSG (ASKA Pharmaceutical Co. Ltd.), followed by an intraperitoneal injection of 300 IU/Kg hCG (ASKA Pharmaceutical Co. Ltd.) 48 h later. Female rats were mated with male rats overnight after hCG injection. The oviducts of female rats with vaginal plugs were removed after euthanasia by CO_2_ and cervical dislocation and oocytes were flushed out from the ampullae with culture medium. Pronuclear-stage embryos were collected and stored in culture medium before introduction of mRNA.

### Introduction of Cas9 mRNA, gRNA and ssODN into intact embryos by the TAKE method

The TAKE method [13] was used to introduce Cas9 mRNA, gRNA and ssODN into intact mouse and rat embryos. This method introduced endonucleases into intact embryos using a super electroporator NEPA 21 (NEPA GENE Co. Ltd., Chiba, Japan). Briefly, pronuclear-stage embryos were placed in a line on the glass chamber between 5mm gap platinum metal plates (CUY520P5, NEPA GENE Co. Ltd.) that were filled with 100 μL PBS containing Cas9 mRNA, gRNA and ssODN at various concentrations. The poring pulse was set to voltage: 225 V, pulse width: 2.5 ms, pulse interval: 50 ms, and number of pulses: +4. The first and second transfer pulse were set to voltage: 20 V, pulse width: 50 ms, pulse interval: 50 ms, and number of pulses: ±5. After electroporation, all embryos were cultured before embryo transfer.

### Embryo transfer

Embryos that developed to the two-cell stage after the introduction of RNA and ssODN were transferred into the oviducts of female surrogate that anesthetized using isoflurane [17]. Females were mated with vasectomised male rats the day before the embryo transfer. The number of offspring that were born naturally was counted at 19–21 days of gestation.

### Analysis of gene editing in offspring

Editing of the targeted gene was analyzed using genomic DNA extracted from blood that was adhered to FTA cards [18]. PCR templates were then prepared by punching out discs from the FTA card in conjunction with Ampdirect Plus buffer (Shimadzu Co., Kyoto, Japan). PCR was performed in a total volume of 15 μL under the following conditions: 1 cycle of 94°C for 3 min; 35 cycles of 94°C for 30 s, 60°C for 1 min and 72°C for 45 s; and 1 cycle of 72°C for 3 min. The final reaction mixture contained 200 μM dNTPs, 1.0 mM MgCl_2_, 0.66 μM of each primer (amplifying the mouse *Il2rg* locus: 234 bp, rat *Il2rg* locus: 292 bp, S1 Table) and 0.4 U Taq DNA polymerase (Thermo Fisher Scientific Inc.). The PCR products were directly sequenced using the BigDye terminator v3.1 cycle sequencing mix and the standard protocol for an Applied Biosystems 3130 DNA Sequencer (Thermo Fisher Scientific Inc.).

### Analysis of germ-line transmission

Certain rat offspring with a one base exchange at the *Il2rg* locus (knock-in) that were derived from the TAKE method were selected. They mated naturally with wild-type male or female rats after they had matured. The edited gene in the offspring of the next generation was then analysed.

### Data analysis

All data were analysed using Chi-squared tests using Yates’ correction for continuity.

## Results

### Introduction of RNA and ssODN into intact mouse pronuclear-stage embryos

Cas9 mRNA, gRNA and ssODN was electroporated into intact mouse pronuclear-stage embryos. The gRNA that targeted mouse *Il2rg* gene was used. Intact mouse pronuclear-stage embryos were electroporated with final concentrations of Cas9 mRNA/gRNA/ssODN of 400/600/300, 200/200/200 and 100/100/100 μg/mL, respectively. After electroporation, the embryos that developed to the two-cell stage were transferred into the oviducts of pseudopregnant female mice.

Of the embryos produced by electroporation with 400/600/300, 200/200/200 and 100/100/100 μg/mL of Cas9 mRNA/gRNA/ssODN, 84, 73 and 98% of embryos survived, respectively. Thereafter, 43, 48 and 24% of the embryos, respectively, developed into offspring, and 67, 31 and 32%, respectively, of these offspring had an edited *Il2rg* locus (knockout). Furthermore, 33, 9 and 18% of the offspring had a one base exchange (knock-in) at the *Il2rg* locus (Table 1).

**Table 1:** Development of mouse embryos with introduced RNAs and ssODN using the TAKE method.

**Cas9 mRNA (μg/mL)**	gRNA (μg/mL)	ssODN (μg/mL)	No. embryos examined	No. (%) of embryos developed to 2-cells^a^	No. (%) of males (M) and females (F)^b^	No. (%) of KO males (M) and females (F)^c^	No. (%) of KI males (M) and females (F)^c^	% (No. of KO/KI offspring/No. embryos examined)
400	600	300	100	84 (84)	M22, F14 (43)^a^	M11, F13 (67)^c^	M4, F8 (33)^e^	24 / 12
200	200	200	100	73 (73)	M21, F14 (48)^a^	M6, F5 (31)^d^	M2, F1 (9)^f^	11 / 3
100	100	100	120	117 (98)	M16, F12 (24)^b^	M5, F4 (32)^d^	M2, F3 (18)	8 / 4

^a^Calculated from the number of embryos examined.

^b^Calculated from the number of embryos developed to two cells.

^c^Calculated from the number of male and female mice.

Significant differences at *P* < 0.05; a *vs*. b, c *vs*. d, e *vs*. f.

### Introduction of RNA and ssODN into intact rat pronuclear-stage embryos

Intact rat pronuclear-stage embryos were electroporated Cas9 mRNA, gRNA and ssODN using the same RNA/ssODN concentration and electrical pulse conditions as the mouse embryos. The gRNA that targeted rat *Il2rg* gene was used. Of the embryos produced by electroporation with 400/600/300, 200/200/200 and 100/100/100 μg/mL of Cas9 mRNA/gRNA/ssODN, 75, 98 and 99% of embryos survived, respectively. Thereafter, 53, 39 and 47%, respectively, of the embryos developed into offspring, and 88, 37 and 39%, respectively, of these offspring showed knockout editing of the *Il2rg* locus (Table 2). Furthermore, 33, 5 and 2%, respectively, of the offspring showed knock-in with one base exchange at the *Il2rg* locus. Germline transmission of the induced mutations was confirmed in the next generation (S2 Table).

**Table 2:** Development of rat embryos with introduced RNA and ssODN using the TAKE method.

**Cas9 mRNA (μg/mL)**	gRNA (μg/mL)	ssODN (μg/mL)	No. embryos examined	No. (%) of embryos developed to 2-cells^a^	No. (%) of males (M) and females (F)^b^	No. (%) of KO males (M) and females (F)^c^	No. (%) of KI males (M) and females (F)^c^	% (No. of KO/KI offspring/No. embryos examined)
400	600	300	60	45 (75)	M14, F10 (53)	M13, F8 (88)^a^	M2, F6 (33)^c^	35 / 13
200	200	200	50	49 (98)	M10, F9 (39)	M1, F6 (37)^b^	F1 (5)	14 / 2
100	100	100	89	88 (99)	M25, F16 (47)	M9, F7 (39)^b^	M1 (2)^d^	18 / 1

^a^Calculated from the number of embryos examined.

^b^Calculated from the number of embryos developed to two cells.

^c^Calculated from the number of male and female rats.

Significant differences at *P* < 0.05; a *vs*. b, c *vs*. d.

## Discussion

Here, we demonstrated the efficient use of the TAKE method to produce knockout and knock-in mice and rats using the CRISPR/Cas system. The mice and rats derived from embryos introduced with 400 μg/mL of Cas9 mRNA, 600 μg/mL of gRNA and 300 μg/mL of ssODN showed editing of the targeted gene as a knockout (67% of mice and 88% or rats) or showed knock-in with one base exchange at the *Il2rg* locus (both 33%), respectively (Tables 1 and 2). The advantage of this method was that the mouse and rat genomes could be easily and quickly edited using same protocol. GM animals are usually generated by microinjection of endonucleases into embryos. Although this has been used routinely as a gold standard technique, the high level of skill and time required has prevented the rapid production of GM animals. Electroporation is an alternative method for introducing exogenous DNA/RNA into embryos. However, the conventional protocol required weakening of the zona pellucida of the embryos by treatment with Tyrode’s acid solution before electroporation for the efficient introduction of DNA [19,20]. Weakening the zona pellucida may have negative effects because the zona pellucida is important for subsequent embryonic development *in vivo* [21,22]. The TAKE method permits the introduction of endonucleases into intact embryos without weakening the zona pellucida. Thus, TAKE could efficiently introduce engineered endonucleases, such as ZFN, TALEN and CRISPR/Cas9 mRNA, into intact embryos using a NEPA 21 electroporator with a three-step electrical pulse system [13]. First electrical step (the poring pulse) makes microscopic holes in the zona pellucida and oolemma. In the second step (the transfer pulse), the initial pulses transfer mRNA into the cytoplasm. In the third step, the polarity-changed second-transfer pulse increases the chance of transferring mRNA into embryos. The TAKE method has dramatically increased the speed of production of GM animals. In previous methods, a high proportion of knockout rats with genome editing were produced by the introduction of ZFN mRNA. However, suitable conditions to generate knockout rats using CRISPR/Cas9 have not been demonstrated. The results of the present study showed that the mice and rats derived from embryos had knockout and knock-in editing of the *Il2rg* locus. Although the success rate of GM animal production depends on the concentration of RNA, animals with edited genomes could be obtained with as little as 100 μg/mL of Cas9 mRNA, 100 μg/mL of gRNA and 100 μg/mL of ssODN using the TAKE method (Tables 1 and 2).

In conclusion, we demonstrated that the TAKE method could be adapted to produce knockout and knock-in mice and rats. This technology has the potential to produce GM animals quickly, easily and successfully without special skills such as conventional pronuclear microinjection method. Furthermore, high success rate lead to reduction the number of females used as oocyte donors, which is applicable the principle of three Rs- replacement, reduction and refinement of animal use [23]. TAKE is also applicable to editing targeted genes of embryos collected from various animals. We believe that the rapid and efficient production of GM animals using TAKE method with CRISPR/Cas9 system will contribute further to our understanding of gene functions and human diseases.

## Supporting Information

S1 Fig Sequence of the mouse (A) and rat (B) *Il2rg* locus. Red capital letters indicate the position of the one base exchange.
(DOCX)

S1 Table Sequences of single-stranded donor oligonucleotides (ssODNs) and primers for the Il2rg locus. (DOCX)

S2 Table Germ-line transmission in rat offspring with an edited *Il2rg* locus derived from the technique for animal knockout system by the electroporation (TAKE) method. (DOCX)
