# Supplementary material for: Simple Genome Editing of Rodent Intact Embryos by Electroporation
Source: PLoS One. 2015 Nov 10;10(11):e0142755. doi: 10.1371/journal.pone.0142755 (PMC4640526; doi:10.1371/journal.pone.0142755)
Supplement: S1 Fig — Red capital letters indicate the position of the one base exchange. (DOCX) [file pone.0142755.s001.docx]

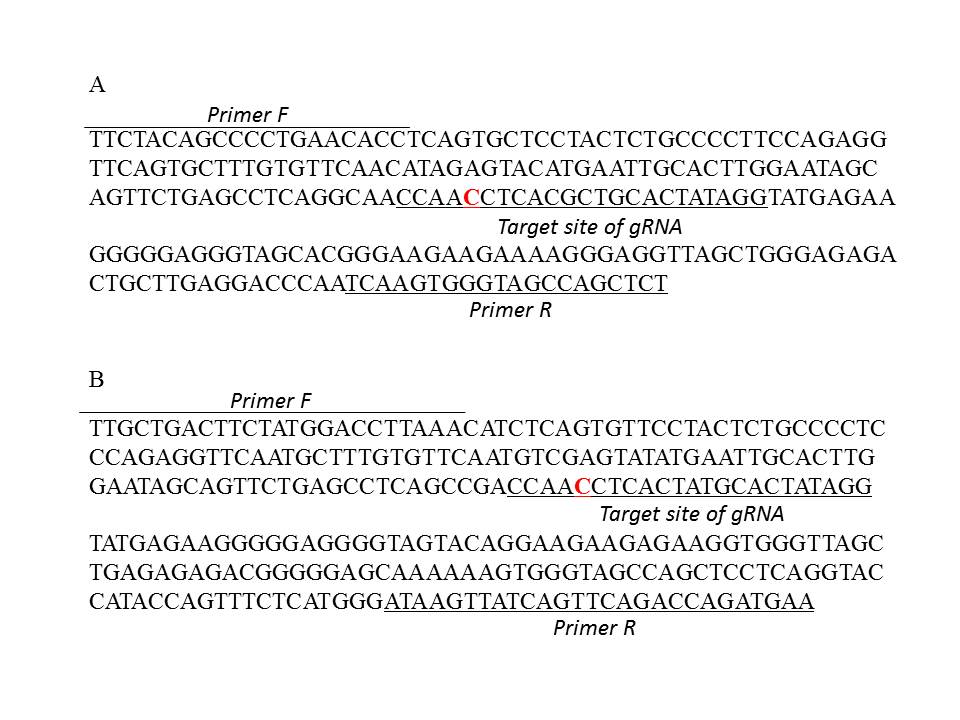


**S1 Fig. Sequence of the mouse (A) and rat (B) *Il2rg* locus.** Red capital letters indicate the position of the one base exchange.
