# Supplementary material for: Simple Genome Editing of Rodent Intact Embryos by Electroporation
Source: PLoS One. 2015 Nov 10;10(11):e0142755. doi: 10.1371/journal.pone.0142755 (PMC4640526; doi:10.1371/journal.pone.0142755)
Supplement: S1 Table — (DOCX) [file pone.0142755.s002.docx]

**S1 Table. Sequences of single-stranded donor oligonucleotides (ssODNs) and primers for the Il2rg locus.**

| Name | Sequences |
| --- | --- |
| Mouse |  |
| ssODN* | TTCAACATAGAGTACATGAATTGCACTTGGAATAGCAGTTCTGAGCCTCAGGCAACCAA**A**CTCACGCTGCACTATAGGTATGAGAAGGGGGAGGGTAGCACGGGAAGAAGAAAAGGGAGG |
| Primer (F) | TTCTACAGCCCCTGAACACC |
| Primer (R) | AGAGCTGGCTACCCACTTGA |
|  |  |
| Rat |  |
| ssODN* | TTCAATGTCGAGTATATGAATTGCACTTGGAATAGCAGTTCTGAGCCTCAGCCGACCAA**A**CTCACTATGCACTATAGGTATGAGAAGGGGGAGGGGTAGTACAGGAAGAAGAGAAGGTGG |
| Primer (F) | TTGCTGACTTCTATGGACCTTAAA |
| Primer (R) | TTCATCTGGTCTGAACTGATAACTTAT |

* Red capital letters indicate the position of the one base exchange (from C to A).
