# Supplementary material for: Simple Genome Editing of Rodent Intact Embryos by Electroporation
Source: PLoS One. 2015 Nov 10;10(11):e0142755. doi: 10.1371/journal.pone.0142755 (PMC4640526; doi:10.1371/journal.pone.0142755)
Supplement: S2 Table — (DOCX) [file pone.0142755.s003.docx]

**S2 Table. Germ-line transmission in rat offspring with an edited *Il2rg* locus derived from the technique for animal knockout system by the electroporation (TAKE) method.**

| Cas9 mRNA/gRNA  /ssODN  (μg/mL) | Males | Females | No. of males (M) and females (F) | No. of males (M) and females (F) with editing targeted gene |
| --- | --- | --- | --- | --- |
| 400/600/300 | Knock-in | Wild type | M7, F5 | F5 |
| 400/600/300 | Wild type | Knock-in | M5, F9 | M5, F4 |
| 200/200/200 | Wild type | Knock-in | M7, F4 | M3, F1 |
| 100/100/100 | Knock-in | Wild type | M7, F7 | F3 |

ssODN; single-stranded donor oligonucleotide
